# The Interplay between TRPM7 and MagT1 in Maintaining Endothelial Magnesium Homeostasis

**DOI:** 10.3390/membranes13030286

**Published:** 2023-02-28

**Authors:** Sara Castiglioni, Laura Locatelli, Giorgia Fedele, Alessandra Cazzaniga, Emil Malucelli, Stefano Iotti, Jeanette A. Maier

**Affiliations:** 1 Department of Biomedical and Clinical Sciences, Università di Milano, 20157 Milano, Italy; 2 Department of Pharmacy and Biotechnology, University of Bologna, 40127 Bologna, Italy; 3 National Institute of Biostructures and Biosystems, 00136 Rome, Italy

**Keywords:** TRPM7, HUVEC, MagT1, magnesium

## Abstract

The transient receptor potential cation channel subfamily M member 7 (TRPM7) is an ubiquitous channel fused to an α-kinase domain involved in magnesium (Mg) transport, and its level of expression has been proposed as a marker of endothelial function. To broaden our present knowledge about the role of TRPM7 in endothelial cells, we generated stable transfected Human Endothelial Cells derived from the Umbilical Vein (HUVEC). TRPM7-silencing HUVEC maintain the actin fibers’ organization and mitochondrial network. They produce reduced amounts of reactive oxygen species and grow faster than controls. Intracellular Mg concentration does not change in TRPM7-silencing or -expressing HUVEC, while some differences emerged when we analyzed intracellular Mg distribution. While the levels of the plasma membrane Mg transporter Solute Carrier family 41 member 1 (SLC41A1) and the mitochondrial channel Mrs2 remain unchanged, the highly selective Magnesium Transporter 1 (MagT1) is upregulated in TRPM7-silencing HUVEC through transcriptional regulation. We propose that the increased amounts of MagT1 grant the maintenance of intracellular Mg concentrations when TRPM7 is not expressed in endothelial cells.

## 1. Introduction

Magnesium (Mg) is important for cardiovascular health, and low-serum Mg is associated with a higher risk of coronary artery disease [1,2]. Preclinical studies have demonstrated that Mg deficiency induces oxidative stress in endothelial cells [3], activating the pro-inflammatory and pro-thrombotic pathways responsible for endothelial dysfunction. Moreover, Mg inhibits lipopolysaccharide-induced inflammation in Human Endothelial Cells derived from the Umbilical Vein (HUVEC) through P2X7 Receptor (P2X7R) blockade and inhibition of the NLR family pyrin domain-containing 3 (NLRP3) inflammasome [4].

Intracellular Mg homeostasis is guaranteed by the presence of several plasma membrane channels and transporters. As previously shown, human endothelial cells express the Transient Receptor Potential Melastatin (TRPM) 7 cation channel [5,6,7], an ubiquitously expressed channel fused to a C-terminal α-kinase domain. The channel is permeable not only to Mg, but also to zinc and calcium. The α-kinase affects the activity of different proteins, among which annexin-1, calpain-2 and phospholipase C2 [8], and the peptides derived from its cleavage bind chromatin-remodeling complexes and epigenetically regulate transcription [9]. In HUVEC, TRPM7 is upregulated by culture in Mg-deficient media or by high glucose through the action of free radicals [6,10], while it is downregulated in cells cultured in media containing high concentrations of Mg (i.e., 5 mM Mg) by the activation of calpains [6]. Moreover, transient TRPM7-silencing stimulates HUVEC proliferation and migration [6] by promoting ERK phosphorylation [7] and also induces nitric oxide synthesis and tubulogenesis in Matrigel [7], to the point that TRPM7 has been proposed as a regulator of endothelial function [6]. Accordingly, high levels of TRPM7 in the aorta of Mg-deficient mice are associated with a pro-thrombotic and pro-inflammatory endothelial phenotype [11]. HUVEC also express the highly selective Mg transporter MagT1, which is not modulated by low extracellular Mg and oxidative stress [12]. Recently, MagT1 has been shown to be also involved in N-glycosylation [13]. It is noteworthy that loss-of-function mutations of MagT1 cause hypomagnesemia within a complex immunodeficiency syndrome [13]. 

At the moment, no data are available in HUVEC about the expression of another plasma membrane Mg transporter, i.e., Solute Carrier family 41 member 1 (SLC41A1). Similarly, no reports investigated the expression of Mrs2, which is located in the inner mitochondrial membrane and is pivotal for the cellular energy status and vulnerability to stress [14].

The aim of this study was to evaluate the interplay among Mg transporters in maintaining Mg homeostasis. To this end, we generated stable transfected TRPM7-silencing HUVEC using the pTRIPZ lentiviral plasmid, which contains the short hairpin RNA (shRNA) targeting the TRPM7 gene, and evaluated the behavior of these cells and the levels of MagT1, SLC41A1 and Mrs2. 

## 2. Materials and Methods

### 2.1. Cell Culture

HUVEC were purchased from the American Type Culture Collection (Manassas, VA, USA) and serially passaged in M199 (Euroclone, Milan, Italy) containing 10% fetal bovine serum (Euroclone, Milan, Italy), 2 mM glutamine (Euroclone, Milan, Italy), 1 mM sodium pyruvate (Sigma-Aldrich, St. Louis, MO, USA), 5 U/mL heparin (Sigma-Aldrich, St. Louis, MO, USA) and 150 µg/mL endothelial cell growth factor supplement (Sigma-Aldrich, St. Louis, MO, USA) on collagen-coated dishes (50 µg/mL) (Sigma-Aldrich, St. Louis, MO, USA). 

### 2.2. Generation of Stably Transfected HUVEC with pTRIPZ Lentiviral Vector Containing TRPM7 shRNA

**Figure 1:**
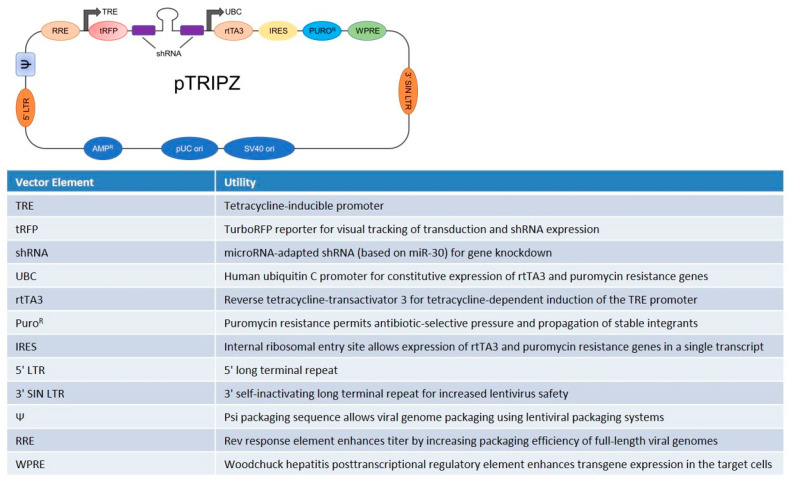
Schematic reproduction and features of the pTRIPZ Inducible Lentiviral shRNA vector.

The lentiviral vector pTRIPZ containing TRPM7 shRNA was purchased from Open Biosystems (Huntsville, AL, USA). The vector pTRIPZ containing a non-silencing shRNA was used as control. The vector map is reported in Figure 1. The pTRIPZ vector is engineered to be Tet-On, providing induced expression of the shRNA in the presence of doxycycline (Sigma-Aldrich, St. Louis, MO, USA). pTRIPZ contains two main components which enable shRNA induction: (1) the tetracycline response element (TRE) and (2) the transactivator. The TRE shows reduced basal expression and tighter binding to the second component, the transactivator. The transactivator, or reverse tetracycline transactivator 3 (rtTA3), binds to the TRE promoter in the presence of doxycycline, thus activating the expression of the shRNA. The plasmids were transfected into HUVEC using Arrest-In Trasfection Reagent (Open Biosystems, Thermo Fisher Scientific, Waltham, MA, USA) according to the manufacturer’s recommendations, and transfected cells were selected using a culture medium containing 0.3 µg/mL puromycin (Sigma-Aldrich, St. Louis, MO, USA). We obtained some stably transfected clones with the pTRIPZ vector containing TRPM7 shRNA (TRPM7 shRNA) or non-silencing shRNA (CTR). To induce shRNA expression, the puromycin resistant cells were treated with 2 µg/mL doxycycline. The TRE promoter drives the expression of the turbo Red Fluorescence Protein (tRFP) reporter gene in addition to the shRNA. Therefore, we initially assessed the induction of shRNA with doxycycline by visualizing the red fluorescence using a FLoid Microscope (Thermo Fisher Scientific, Waltham, MA, USA). 

### 2.3. Cell Proliferation and Reactive Oxygen Species Production

10^4^ TRPM7-silencing/-expressing HUVEC/cm^2^ were seeded in a 24-well plate and were treated with doxycycline (2 µg/mL) (Sigma-Aldrich, St. Louis, MO, USA) on the following day for an additional 24, 48 or 72 h. The cells were then trypsinized, stained with 0.4% trypan blue solution and counted using a LUNA II automated cell counter (Logos Biosystems, Dongan-gu Anyang-si, Gyeonggi-do, Republic of Korea).

Reactive oxygen species (ROS) production was quantified using 2′-7′-dichlorofluorescein diacetate (DCFH, Thermo Fisher Scientific, Waltham, MA, USA). Stable transfected TRPM7-silencing/-expressing HUVEC were cultured into black-bottomed 96-well plates (Greiner BioOne, Kremsmünster, Austria), treated with 2 µg/mL of doxycycline for 24 h and exposed for 30 min to 20 µM DCFH solution [15]. The emission at 529 nm of the DCFH dye was monitored using Varioskan Lux (Thermo Fisher Scientific, Waltham, MA, USA), and the results were normalized on nuclei stained with 4′,6-Diamidine-2′-phenylindole dihydrochloride (DAPI, Sigma-Aldrich, St. Louis, MO, USA). The results are the means of three independent experiments performed in triplicate ± standard deviation (SD). 

### 2.4. Western Blot

The cells were lysed in 10 mM Tris-HCl (pH 7.4) containing 3 mM MgCl_2_, 10 mM NaCl, 0.1% SDS, 0.1% Triton X-100, 0.5 mM EDTA and protease inhibitors, separated on SDS-PAGE and transferred to nitrocellulose sheets at 400 mA for 2 h at 4 °C. Western blot analysis was performed using antibodies against TRPM7 (Bethyl, Montgomery, AL, USA), MagT1 (Abcam, Cambridge, UK), SLC41A1, Mrs2, cyclophilin D (CypD), thioredoxin-interacting protein (TXNIP), paraoxonase 2 (PON2) and superoxide dismutase 2 (SOD2) (Thermo Fisher Scientific, Waltham, MA, USA), heat shock protein (Hsp) 27 (Cell Signaling Technology, Danvers, MA, USA), Hsp70 and actin (Santa Cruz Biotechnology, Dallas, TX, USA). After extensive washing, secondary antibodies labeled with horseradish peroxidase (GE Healthcare, Waukesha, WI, USA) were used. Immunoreactive proteins were detected by the SuperSignal Chemiluminescence Kit (Thermo Fisher Scientific, Waltham, MA, USA). All the experiments were performed three times, and a representative blot is shown. Densitometry of the bands was performed with the software ImageLab (Bio-Rad, Hercules, CA, USA) on three blots ± (SD).

### 2.5. Quantification of Total Intracellular Mg Concentration

Total Mg content was measured using the fluorescent chemosensor DCHQ5 as described in [16,17,18] in *TRPM7-*silencing/-expressing HUVEC. Fluorescence intensities were acquired at 510 nm using Varioskan Lux. Mg concentrations of the samples were obtained by the interpolation of their fluorescence with the standard curve obtained using MgSO_4_. Results were normalized on cell numbers. All the experiments were performed three times in triplicate ± SD.

### 2.6. Real-Time PCR 

Total RNA was extracted from TRPM7-silencing/-expressing HUVEC by the PureLink RNA Mini kit (Thermo Fisher Scientific, Waltham, MA, USA). Single-stranded cDNA was synthesized from 0.2 μg RNA in a 20 μL final volume using a High-Capacity cDNA Reverse Transcription Kit, with RNase inhibitor (Thermo Fisher Scientific, Waltham, MA, USA) according to the manufacturer’s instructions. Real-time PCR was performed using the CFX96 Real-Time PCR System instrument (Biorad, Hercules, CA, USA) using TaqMan Gene Expression Assays (Life Technologies, Thermo Fisher Scientific, Waltham, MA, USA): *MagT1* (Hs00997540_m1). The housekeeping gene *GAPDH* (Hs99999905_m1) was used as an internal reference gene. Relative changes in gene expression were analyzed by the 2^−ΔΔCt^ method [19]. The experiments were repeated three times in triplicate ± SD.

### 2.7. Confocal Microscopy

TRPM7-silencing/-expressing HUVEC were fixed in phosphate-buffered saline containing 4% paraformaldehyde and 2% sucrose (pH 7.6), incubated with anti-cyclophilin D overnight at 4 °C and stained with Alexa Fluor 488 secondary antibody (Thermo Fisher Scientific, Waltham, MA, USA). We used tetramethylrhodamine (TRITC)-labeled phalloidin to visualize the cytoskeleton and DAPI (Sigma-Aldrich, St. Louis, MO, USA) to stain the nuclei. Finally, the samples were mounted with ProLong Gold Antifade Mountant (Thermo Fisher Scientific, Waltham, MA, USA), and images were acquired using a 20× objective by a SP8 Leica confocal microscope.

### 2.8. Atomic Force Microscopy Measurements

Cell thickness maps were collected using a Digital Instruments D3100 Atomic Force Microscope (AFM) equipped with a Nanoscope IIIa controller. Measurements were carried out in air in Tapping Mode at a resonance frequency of about 260 kHz by use of monolithic silicon tips with an apex curvature radius in the 5^−10^ nm range and a typical force constant of ∼40 N·m^−1^. The typical square scan size used was on the order of 50 μm × 50 μm, and the matrix resolution in pixels was 512 × 512. For further detail on the AFM measurements see [20]. The cell boundaries were automatically delineated from the AFM thickness maps, exploiting the intrinsically lower noise of AFM technique with respect to X-ray based imaging, which allows for a more precise definition of the cell shape. The masks of the cells obtained by the AFM segmentation have been used to mask out all the areas outside the cell (black pixel in the images). Moreover, the inverse of this mask has been utilized to calculate the background of all the maps.

### 2.9. X-ray Fluorescence Microscopy and Scanning Transmission Microscopy Measurements

The X-ray fluorescence microscopy (XRFM) and scanning transmission X-ray microscopy (STXM) measurements were carried out at the beamline TwinMic [21] at Elettra Synchrotron (Trieste, Italy). The TwinMic instrument combines scanning and full-field imaging in a single instrument with a manifold of contrast techniques and spectroscopies. A Fresnel zone plate focused the incoming beam (1475 eV), monochromatized by a plane grating mono-chromator, to a circular spot of about 600 nm in diameter. Five STXM images were acquired on whole cells with 25 ms dwell time per step, with a step size of 500 nm. Next, XRFM were carried out with a range of 6−8 s dwell time per pixel depending on the cell size. The total acquisition time, for each XRFM scan, was in the range of 7–9 h (field of view of at least 20 × 20 μm^2^; spatial resolution of 500 nm).

### 2.10. Elemental Quantification

The single cells’ elemental quantification was based on the method proposed by Malucelli et al. [20]. Briefly, the mass fraction *W* was calculated, merging the information coming from XRFM, STXM and AFM using the fundamental parameter equation [22]:

(1) $$W\left(i\right)=\frac{R}{\left[\rho V\right]\left[Y\right]F_{p}}$$

where *V* is the volume measured by AFM; *ρ* is the density calculated by the Beer–Lambert law (using cell thickness data obtained by AFM analysis) and by considering a given cell composition taken from the literature [23]. *R* is the contribution to the total counts for that specific fluorescence line derived by the analysis of X-ray fluorescence spectra using PyMCA software [24]. *Y* is a set of constants; some are linked to the element measured such as the fluorescence yield, the transition probability and the photon electron cross-section, while others are linked to the characteristics of the beamline such as the detector efficiency and solid angle seen by the detector. The detector efficiency has been evaluated examining two standards: one constituted by a Mg film 200 nm-thick covered by an Au film of 50 nm; and the other constituted by a bare 200 nm-thick Si_3_N_4_ window, equal to those used as substrate for our samples. Finally, *Fp* is the correction factor for the self-absorption of both incident beam and fluorescence radiation, calculated using an ad hoc homemade algorithm [25].

The elemental molar concentration was calculated using the following equation:

(2) $$M\left(i\right)=\frac{W\left(i\right)\times \rho }{A_{i}}$$

where *Ai* is the atomic weight of the *i*th element. For further information on the quantification method, see Malucelli et al. [20].

### 2.11. Statistical Analysis

Statistical significance was determined using a *t*-test with Welch’s correction and was set as follows: * *p* < 0.05, ** *p* < 0.01, *** *p* < 0.001.

## 3. Results

### 3.1. Generation of TRPM7-Silencing HUVEC

To obtain stable transfectants silencing TRPM7, we used the pTRIPZ lentiviral, which was engineered to be Tet-On. The plasmid was designed to contain the shRNA-targeting TRPM7 gene. The vector pTRIPZ containing a non-silencing shRNA was used as control. The Tet-inducible technology enables TRPM7 shRNA or non-silencing shRNA to be expressed only in the presence of doxycycline, with the aim of obtaining homogeneous populations of TRPM7-silencing cells. 

**Figure 2:**
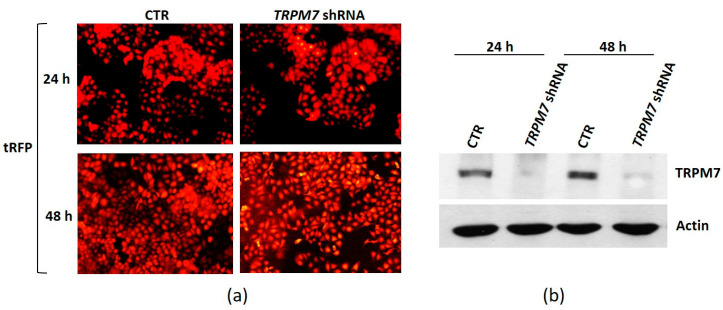
Generation of TRPM7-silencing HUVEC. The lentiviral vector pTRIPZ containing TRPM7 shRNA or non-silencing shRNA was transfected in HUVEC. The transfected cells were cultured in the presence of doxycycline for 24 and 48 h to induce TRPM7 shRNA (*TRPM7 *shRNA) or non-silencing shRNA (CTR) expression. (**a**) tRFP fluorescence was visualized in TRPM7 shRNA and non-silencing shRNA cells (CTR). (**b**) The levels of TRPM7 were evaluated by Western blot. Actin was used as control of loading. A representative blot is shown.

After doxycycline treatment, both the TRPM7 shRNA (*TRPM7 *shRNA) and the non-silencing shRNA (CTR) clones show a high expression of the tRFP reporter gene as detected by fluorescence microscopy (Figure 2a). By Western blot, only the TRPM7 shRNA clone significantly downmodulates TRPM7 (Figure 2b). 

### 3.2. TRPM7 Silencing Decreases ROS Production in HUVEC

**Figure 3:**
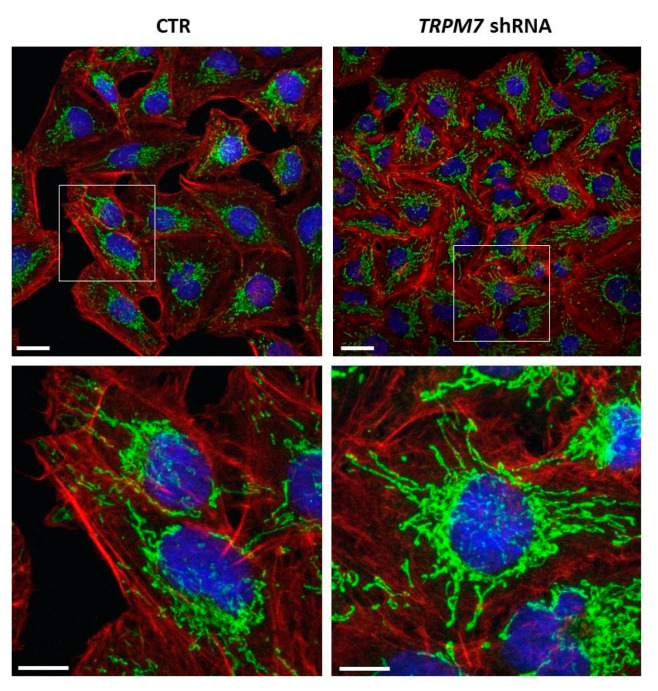
Analysis of cell morphology and mitochondria in TRPM7-silencing/-expressing HUVEC (TRPM7 shRNA and CTR, respectively). TRPM7-silencing/-expressing HUVEC were observed by confocal microscopy after TRITC-phalloidin (red), cyclophilin D (green) and DAPI (blue) staining. The white squares in the upper images were enlarged in the lower panel. Scale bar: 20 µm.

We have previously demonstrated that TRPM7 has a role in the modulation of endothelial behavior [5,6]. To better characterize TRPM7-silencing vs. TRPM7-expressing HUVEC, we analyzed cell morphology by staining the actin cytoskeleton with phalloidin. By fluorescence microscopy, we show that the cells maintain the same morphology, with similar organization of actin fibers (Figure 3, red staining). We then evaluated mitochondria, which not only have a central role in energy metabolism, but also control stress responses [26]. Immunofluorescence using antibodies against cyclophilin D shows that mitochondria are elongated and form a complex mitochondrial network both in TRPM7-silencing and TRPM7-expressing HUVEC (Figure 3, green staining).

**Figure 4:**
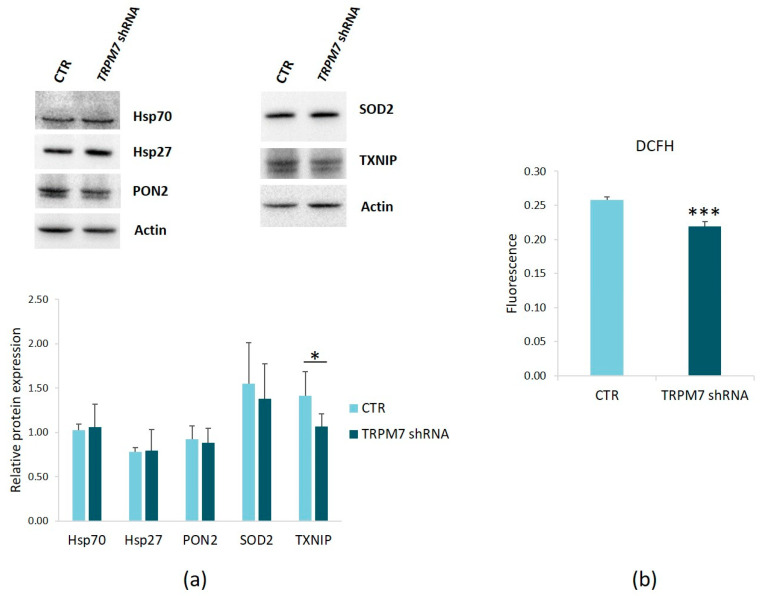
Characterization of TRPM7-silencing HUVEC. (**a**) Protein lysates from TRPM7-silencing/-expressing HUVEC (TRPM7 shRNA and CTR, respectively) were analyzed by Western blots using antibodies against Hsp70, Hsp27, PON2, SOD2 and TXNIP. Anti-actin antibodies were used as control of equal loading. A representative blot of three independent experiments is shown (**upper panel**). Densitometry of the bands was performed on three blots with the software ImageLab (**lower panel**). (**b**) ROS production was quantified using DCFH and normalized on nuclei stained with DAPI. * *p* ≤ 0.05, *** *p* ≤ 0.001.

We also investigated the levels of some stress proteins in TRPM7-silencing/-expressing HUVEC. We demonstrate that the stress proteins Hsp70 and Hsp27 and the antioxidant enzymes PON2 and SOD2 were not significantly altered (Figure 4a), whereas the pro-oxidant TXNIP was downregulated in TRPM7-silencing HUVEC (Figure 4a). Accordingly, TRPM7-silencing decreased ROS production (Figure 4b).

### 3.3. TRPM7 Silencing Increases HUVEC Proliferation

**Figure 5:**
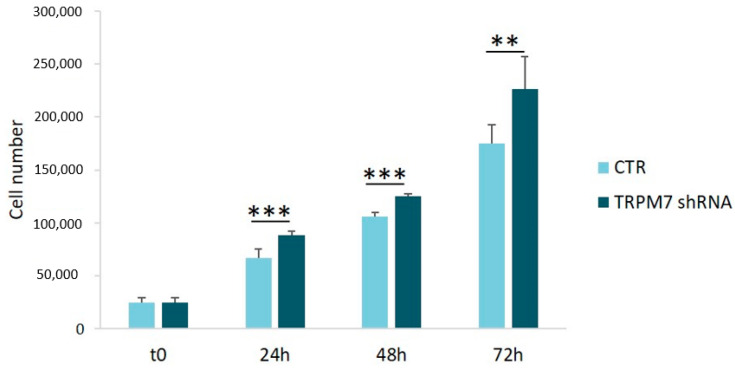
Cell proliferation in TRPM7-silencing HUVEC. TRPM7-silencing/-expressing HUVEC (TRPM7 shRNA and CTR, respectively) were counted with an automated cell counter after 24, 48 and 72 h of treatment with doxycycline. Cell numbers were expressed as the mean ± SD. ** *p* ≤ 0.01, *** *p* ≤ 0.001.

Transient TRPM7-silencing is associated with an increase in HUVEC proliferation, and this is a unique behavior in the landscape of mammalian cells [5]. We counted the cells after 24, 48 and 72 h and confirmed the accelerated growth of stable TRPM7-silencing HUVEC vs. their controls (Figure 5).

### 3.4. TRPM7 Silencing Does Not Modulate Total Mg 

**Figure 6:**
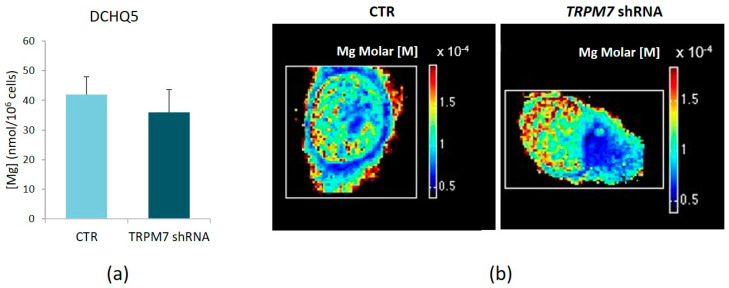
Total Mg concentration and distribution in TRPM7-silencing HUVEC. (**a**) Total Mg in TRPM7-silencing/-expressing HUVEC (TRPM7 shRNA and CTR, respectively) was measured using the fluorescent chemosensor DCHQ5. Data were normalized on cell numbers and expressed as the mean ± SD. (**b**) Elemental nanoscale maps of molar concentration of Mg obtained by single cells analysis is reported. A representative image is shown.

Since TRPM7 is an important regulator of Mg homeostasis and a correct Mg homeostasis is fundamental for endothelial function, we analyzed total intracellular Mg concentration using the fluorescent probe DCHQ5 and found no significant differences between TRPM7-silencing and TRPM7-expressing HUVEC (Figure 6a). 

To investigate whether TRPM7-silencing HUVEC exhibit a different elemental distribution compared to the controls, we combined XRFM, STXM and AFM and obtained a map of Mg concentration distribution (Figure 6b). The TRPM7-silencing cells show a more clustered intracellular Mg concentration distribution, as reported in Figure 6b, in comparison to CTR, where Mg is more homogeneously distributed within the cytosol and is also visualized in the plasma membrane.

### 3.5. TRPM7 Silencing Modulates the Levels of MagT1

**Figure 7:**
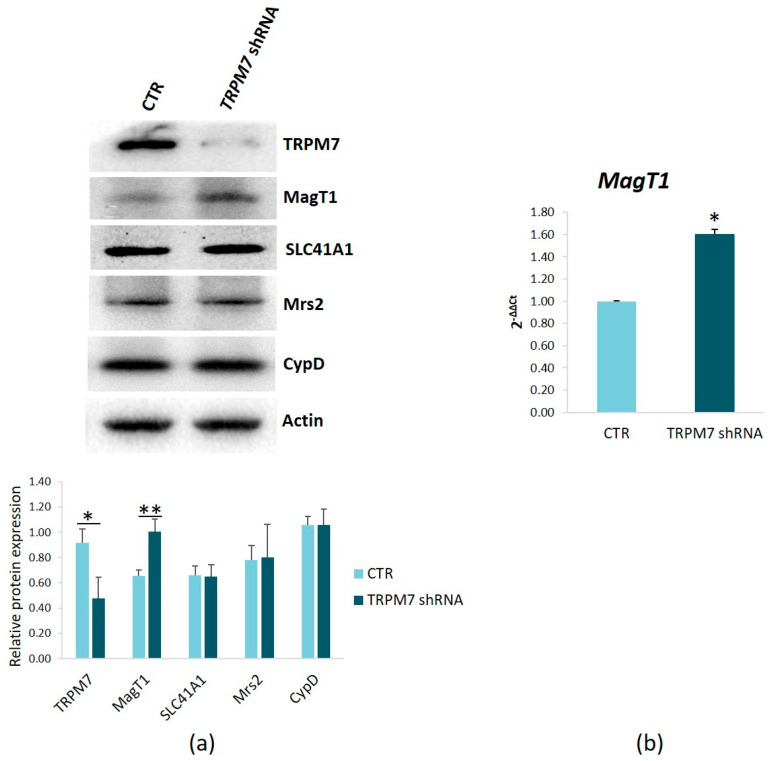
Expression of Mg transporters/channels in TRPM7-silencing HUVEC. (**a**) Protein lysates from TRPM7-silencing/-expressing HUVEC (TRPM7 shRNA and CTR, respectively) were analyzed by Western blots using antibodies against TRPM7, MagT1, SLC41A1 and Mrs2. Anti-actin antibodies were used as controls of equal loading for TRPM7, MagT1 and SLC41A1, while Mrs2 was normalized on CypD. A representative blot of three independent experiments is shown (**upper panel**). Densitometry of the bands on three different blots was performed with the software ImageLab (**lower panel**). (**b**) Real-Time PCR was performed on RNA extracted from TRPM7-silencing/-expressing HUVEC. Primers designed on *MagT1* sequence were used. Data are expressed as the mean ± SD. * *p* ≤ 0.05, ** *p* ≤ 0.01.

To investigate why intracellular Mg remains unchanged despite TRPM7-silencing, we analyzed the total amounts of other Mg channels/transporters. Figure 7a shows that TRPM7-silencing is associated with the upregulation of the Mg transporter MagT1, whereas SLC41A1 and the mitochondrial Mg channel Mrs2 are not modulated. We also analyzed the expression of TRPM6 and, in agremeent with previous results [27], we found that TRPM6 is not expressed in TRPM7-silencing/-expressing HUVEC. To obtain insights into the mechanisms involved in the increase of MagT1, we performed a Real-Time PCR. We found MagT1 transcript overexpressed in TRPM7-silencing cells (Figure 7b).

## 4. Discussion

Mg is fundamental for endothelial health because it is essential for most metabolic pathways and exerts anti-oxidant, anti-inflammatory and anti-thrombotic activities. At the cellular level, Mg homeostasis is maintained by several transporters and channels. Here, we show that HUVEC, widely utilized as an experimental model of macrovascular endothelial cells, express TRPM7, MagT1 and SLC41A1 as well as the mitochondrial transporter Mrs2, whereas TRPM6, mainly implicated in systemic Mg homeostasis through its renal and intestinal action [28], is undetectable. More importantly, we show that TRPM7-silencing results in the upregulation of MagT1, which guarantees the maintenance of physiological intracellular Mg concentrations. The inverse relation between TRPM7 and MagT1 has been reported in different cell types. Colon cancer LoVo cells show high levels of TRPM7 and low amounts of MagT1, while the opposite is true in their doxorubicin-resistant counterpart [29]. In differentiated murine C2C12 myoblasts, TRPM7 levels are reduced and MagT1 levels are increased when compared to undifferentiated cells [30]. At the moment, the molecular mechanisms involved are elusive. Here, we found that TRPM7-silencing HUVEC overexpress *MagT1* transcripts, thus indicating a transcriptional regulation of this transporter in our experimental conditions. Krapivinsky et al. have demonstrated that fragments derived from the proteolytic cleavage of the TRPM7 kinase domain reach the nucleus, where they bind to chromatin components and phosphorylate histones [9]. We hypothesize that the absence of TRPM7 and, therefore, the lack of its nuclear translocated fragments might be involved in affecting the expression of MagT1. It is also feasible that TRPM7-silencing reshapes intracellular signaling pathways, thereby modulating gene expression and cell performance.

We propose that the upregulation of MagT1 explains why the intracellular Mg concentrations do not change in stable transfected TRPM7-silencing HUVEC. We also examined Mg distribution by single cell analysis combining XRFM, STXM and AFM. In agreement with previous studies [17], TRPM7-silencing cells are more heterogeneous in Mg distribution than control cells. This outcome opens the speculation that TRPM7-silencing may introduce some degree of variability, considering that silencing an ion channel may lead to partial metabolic changes in cell physiology.

Stable transfected TRPM7-silencing HUVEC proliferate faster than controls, whereas most mammalian cells, including human microvascular endothelial cells [5], are growth-inhibited in the absence of TRPM7. These results confirm the unique behavior of HUVEC, as previously reported after transient TRPM7-silencing [6]. Here, we also show that TRPM7-silencing HUVEC are not stressed as no upregulation of Hsp70 or Hsp27 occurs. Moreover, TRPM7-silencing HUVEC produce less ROS than controls, and this is associated with a decrease in the pro-oxidant TXNIP. A similar behavior coupling reduced TXNIP levels and decreased ROS was reported in LoVo cells resistant to doxorubicin [29]. On the contrary, oxidative stress generated by culturing HUVEC in Mg-deficient media or by exposing them to H_2_O_2_ or high concentrations of D-glucose upregulates TRPM7 [6,12]. In agreement with our results, ischemia-induced oxidative stress upregulates neuronal TRPM7 in mice, and this contributes to cell death. In conditional TRPM7 knockout animals, the suppression of TRPM7 reduces neuronal death and tissue damage and fosters functional recovery [31]. Similarly, spinal cord injury in rats upregulates TRPM7 through oxidant species, and the inhibition of TRPM7 reduces cell death, in part by preserving the blood–spinal cord barrier [32]. In cardiomyocytes exposed to hypoxia-reoxygenation, miR-129-5p, which targets TRPM7, inhibits ROS production and attenuates cell death [33]. All these data indicate the strict relation between TRPM7 levels and free radicals.

We are aware of several limitations of our study. First, we did not evaluate the transporters’ activity, an issue that should be taken into account in the future. Second, we did not investigate whether HUVEC express the cyclin M (CNNM) transporters, which regulate Mg efflux but also interact with TRPM7 and control its channel function in non-polarized cells [34]. More studies are necessary to characterize CNNMs in the endothelium.

## 5. Conclusions 

Our results indicate that Mg homeostasis is maintained in TRPM7-silencing HUVEC through the upregulation of MagT1. They also point to TRPM7 as a contributor to endothelial health and support our claim that its upregulation is an indicator of endothelial dysfunction. 

## Data Availability

The data presented in this study are openly available in Dataverse through the following link: https://dataverse.unimi.it/dataverse/TRPM7silencingHUVEC, accessed on 29 January 2023.
